# Inhibition of Fungal Growth and Induction of a Novel Volatilome in Response to *Chromobacterium vaccinii* Volatile Organic Compounds

**DOI:** 10.3389/fmicb.2020.01035

**Published:** 2020-05-20

**Authors:** Ghazal Ebadzadsahrai, Emily A. Higgins Keppler, Scott D. Soby, Heather D. Bean

**Affiliations:** ^1^College of Science, Engineering and Technology, Grand Canyon University, Phoenix, AZ, United States; ^2^School of Life Sciences, Arizona State University, Tempe, AZ, United States; ^3^Center for Fundamental and Applied Microbiomics, The Biodesign Institute, Tempe, AZ, United States; ^4^Biomedical Sciences Program, College of Graduate Studies, Midwestern University, Glendale, AZ, United States; ^5^College of Veterinary Medicine, Midwestern University, Glendale, AZ, United States

**Keywords:** fungal pathogen antagonism, volatile organic compound (VOC), inter-kingdom communication, semiochemical communication, quorum sensing (QS), emergent properties

## Abstract

The study of chemical bioactivity in the rhizosphere has recently broadened to include microbial metabolites, and their roles in niche construction and competition via growth promotion, growth inhibition, and toxicity. Several prior studies have identified bacteria that produce volatile organic compounds (VOCs) with antifungal activities, indicating their potential use as biocontrol organisms to suppress phytopathogenic fungi and reduce agricultural losses. We sought to expand the roster of soil bacteria with known antifungal VOCs by testing bacterial isolates from wild and cultivated cranberry bog soils for VOCs that inhibit the growth of four common fungal and oomycete plant pathogens, and *Trichoderma* sp. Twenty one of the screened isolates inhibited the growth of at least one fungus by the production of VOCs, and isolates of *Chromobacterium vaccinii* had broad antifungal VOC activity, with growth inhibition over 90% for some fungi. Fungi exposed to *C. vaccinii* VOCs had extensive morphological abnormalities such as swollen hyphal cells, vacuolar depositions, and cell wall alterations. Quorum-insensitive *cviR*^−^ mutants of *C. vaccinii* were significantly less fungistatic, indicating a role for quorum regulation in the production of antifungal VOCs. We collected and characterized VOCs from co-cultivation assays of *Phoma* sp. exposed to wild-type *C. vaccinii* MWU328, and its *cviR*^−^ mutant using stir bar sorptive extraction and comprehensive two-dimensional gas chromatography—time-of-flight mass spectrometry (SBSE-GC × GC-TOFMS). We detected 53 VOCs that differ significantly in abundance between microbial cultures and media controls, including four candidate quorum-regulated fungistatic VOCs produced by *C. vaccinii*. Importantly, the metabolomes of the bacterial-fungal co-cultures were not the sum of the monoculture VOCs, an emergent property of their VOC-mediated interactions. These data suggest semiochemical feedback loops between microbes that have co-evolved for sensing and responding to exogenous VOCs.

## Introduction

The health of any soil, and therefore the productivity of the plants growing in it, is dependent on the activity and interactions of the microbes that are present (Berendsen et al., 2012; Xiong et al., 2017; Qiao et al., 2019). Culture-independent data have revealed the depth and diversity of microbial populations in the soil, rhizosphere, and phylosphere (Zou et al., 2007; Wenke et al., 2010; Blom et al., 2011; Bailly and Weisskopf, 2012; Farag et al., 2013; De Vrieze et al., 2015; Giorgio et al., 2015; Schmidt et al., 2016; Mülner et al., 2019), and there is considerable evidence that the host plant plays a role in directing the composition of those populations (Sharifi and Ryu, 2018). Additionally, microbial competition for nutritional and niche resources in these environments results in a form of chemical warfare that ostensibly facilitates the overall co-existence of bacteria and fungi in these environments (Paulitz and Bélanger, 2001; Minuto et al., 2006; Ushio et al., 2013; Sharifi and Ryu, 2018).

The full extent of the nature and influence of the compounds that serve as a means of intra- and inter-kingdom communication among and between plant-associated microbial communities is now being explored (Zhang et al., 2017; Jansson and Hofmockel, 2018). Some microbial secondary metabolites, including antibacterial and antifungal compounds, toxins and bio-surfactants (Raaijmakers et al., 2002), are water soluble and therefore diffusible within the soil/water matrix. Soluble compounds can act as semiochemical signals within the soil if there is a continuous liquid interface connecting microbial communities, and if distances are not too great (Westhoff et al., 2017). In contrast, volatile compounds can act through discontinuous systems, or at greater distances (Barr, 1976; Stotzky et al., 1976; Effmert et al., 2012). There is a long history of observations indicating that plant and soil-associated bacteria produce inorganic and organic volatile compounds with antifungal activity (Dobbs and Hinson, 1953; Barr, 1976; Zygadlo et al., 1994; Zou et al., 2007; Effmert et al., 2012; Audrain et al., 2015; De Vrieze et al., 2015; Schmidt et al., 2016; Mülner et al., 2019), and that fungi produce antibacterial volatile organic compounds (VOCs) (Effmert et al., 2012; Werner et al., 2016). Over the past two decades, investigations into the production and biological activities of VOCs have resulted in a large catalog of compounds that are synthesized by soil microorganisms, often in intriguingly complex and dynamic combinations (Mackie and Wheatley, 1999; Fernando et al., 2005; Zou et al., 2007; Korpi et al., 2009; Insam and Seewald, 2010; Effmert et al., 2012; Penuelas and Terradas, 2014; Lemfack et al., 2017; Rajer et al., 2017; Schulz-Bohm et al., 2017; Yuan et al., 2017), though little is yet known about the semiochemical interactions that are mediated by VOCs, nor how those signals are transduced.

Recently we have begun using Cranberry (*Vaccinium macrocarpon* Ait.; Ericaceae) as a model system for microbial interactions because it grows in bogs with relatively open, sandy soil architecture (Eck, 1990), which allows for the moderately free movement of bacteria through the soil (Soby and Bergman, 1983); the geographic proximity of cultivated and wild plants; and its importance as a cash crop in several states and provinces. Cranberry bogs are exceptionally acidic (pH ~3.6–4.3), high iron, low-nutrient environments (Gorham, 1991; Keddy, 2010), therefore, the complex multi-species microbial networks in the soil and rhizosphere that determine the health of these plants have adapted to a set of conditions that are in many respects unlike more familiar agronomically important crops. Cultivated cranberry is susceptible to a number of fungal diseases during its growth and development that can cause significant economic losses through reduced yields and postharvest rot (Oudemans et al., 1998; Oudemans, 1999; Wells-Hansen and Mcmanus, 2017). Cranberry fruit rot is caused by a complex of several fungal species, including *Phoma* sp. (Polashock et al., 2017), *Coleophoma empetri* (Rostr.) Petr., and *Colletotrichum acutatum* J. H. Simmonds (Halsted, 1889; Stevens, 1924; Oudemans et al., 1998; Polashock et al., 2017; Wells-Hansen and Mcmanus, 2017). *Phytophthora cinnamomi* is an important soil-associated oomycete that causes cranberry dieback disorder in cultivated cranberry bogs (Caruso and Wilcox, 1990; Caruso, 2000). These fungi can act separately or in concert to destroy up to 100% of annual yields in the absence of fungicide controls (Oudemans et al., 1998). In contrast, little fruit rot disease is evident in wild cranberry stands (unpublished observations), which may be due to differences in the soil and rhizospheric microbiomes of wild vs. cultivated bogs. This has prompted us to begin exploring the antifungal properties of the plant- and soil-associated bacteria of cranberry bogs.

The objective of this study was to identify cranberry bog soil bacteria that produce antifungal VOCs, and to examine their effects on fungal growth and development. After prescreening for fungal inhibition in a co-culture agar diffusion assay, we used a system that allowed gas exchange while prohibiting the exchange of soluble metabolites to test co-cultures of 21 isolates of bacteria from wild and cultivated cranberry bogs with common cranberry fungal and oomycete pathogens and one biocontrol fungus. Of the bacteria we tested, we found that the VOCs produced by *Chromobacterium vaccinii* generated the highest degree of fungal growth inhibition. We further characterized the chemical compositions of the inhibitory VOCs, their quorum regulation in *C. vaccinii*, and the morphological and chemical responses of *Phoma* sp. upon exposure.

## Materials and Methods

### Microorganisms and Culture Conditions

Bacteria used in this study were isolated from soil and plant samples collected from wild cranberry bogs in the Cape Cod National Seashore (CCNS) in Truro and Provincetown, MA, USA, and from cultivated bogs maintained by The University of Massachusetts and commercial growers in Plymouth County, MA, USA, as previously described (Soby et al., 2013). A multiyear and multiseason sampling protocol has been employed, starting in 2009, to characterize the microbiota of wild and cultivated cranberry bogs. We have used culture-independent as well as the culture-dependent strategy outlined in this manuscript and elsewhere (see, for example, Ebadzadsahrai and Soby, 2019). In this work we prescreened 68 bacterial isolates from both wild and cultivated bog soils for antifungal activity against *Trichoderma* sp., *Phoma* sp., and *Colletotrichum* sp. using the agar-diffusion assay (Grover and Moore, 1962), and isolates that visually inhibited the growth of any one of the fungi were selected for use in this study. Supplementary Table 1 describes the bacterial and fungal isolates used in this study.

Isolates were identified to at least the genus level by 16S rRNA gene sequencing using 27F and 1525R primers (Nicholson et al., 1994; Frank et al., 2008). *Chromobacterium vaccinii* strains were isolated from wild (MWU205) and cultivated (MWU328 and MWU300) cranberry bog soils in Massachusetts, and characterized as a new species (Soby et al., 2013). Spontaneous quorum sensing (QS) null mutants lacking a functional acyl-homoserine lactone receptor (CviR^−^; MWU205W, MWU300W, and MWU328W), and are therefore unable to produce the purple pigment violacein (Mcclean et al., 1997a,b), were isolated as white colonies during routine culture. *Chromobacterium subtsugae* MWU12-2387 and MWU13-2521 were isolated from wild cranberry bogs in the CCNS and identified using molecular phylogeny of the 16S rRNA gene sequences (Supplementary Figure 1). *Pseudomonas chlororaphis* 30-84 was obtained from the Pierson lab at Texas A&M (Yu et al., 2018). *Trichoderma* sp. MWU14-9201 was isolated from wild bog soil, and the berry pathogens *Phoma* sp. MWU-UMCS9302, *Colletotrichum* sp. MWU-UMCS9301, and *Coleophoma* sp. MWU-UMCS9305, were obtained from the University of Massachusetts Cranberry Station. Fungal identification was based on micro-morphological features and verified by 18S and ITS sequencing using the primers NS1 and NS6, and ITS1 and ITS4, respectively (White et al., 1990). *Phytophthora cinnamomi* R001 was provided by the USDA*/*ARS Corvallis, OR (Supplementary Table 1).

All bacterial isolates used for experiments were recovered from −80°C glycerol stocks, plated on Kings Medium B (KMB) agar (King et al., 1954) and grown overnight at 25°C before use. The fungi and *P. cinnamomi* were pre-cultured on Potato Dextrose Agar (PDA) plates and incubated for 6 days at 25°C before assays were performed.

### Antifungal VOC Activity

Twenty one bacterial isolates from 13 genera and three QS mutants of *C. vaccinii* were tested for effects of bacterial volatile organic compounds (bVOCs) on fungal growth and hyphal development of *Trichoderma* sp. MWU14-9201, *Phoma* sp. MWU-UMCS9302, *Colletotrichum* sp. MWU-UMCS9301, *Coleophoma* sp. MWU-UMCS9305, and *P. cinnamomi* R001. The QS mutants of *C. vaccinii* were included to determine if antifungal activity is dependent on quorum sensing. Fungal isolates were grown on PDA at room temperature (25–26°C) for 24 h for *Trichoderma* sp., *Phoma* sp., and *P. cinnamomi*, and 96 h for *Colletotrichum* sp. and *Coleophoma* sp. to accommodate slower growth (Chaurasia et al., 2005), after which 0.5 × 0.5 cm mycelial blocks were harvested from the growing edge of the fungal colonies. Bacterial isolates were grown at 25°C on KMB overnight, then re-streaked on KMB and grown at 25°C for 72 h, after which the lid of the bacterial plate was replaced by a ‘bottom' petri dish plate containing PDA inoculated with a mycelial agar block. This arrangement, which we call a sandwich plate, allows free gas exchange between the fungus and bacterium but no physical contact or exchange of non-volatile metabolites. The plates were joined using sealing tape (Petri-Seal™ Stretch tape, RPI Corp) to contain VOCs and to hold the two petri dish bottoms together. Fungal growth controls were prepared in the same way but without bacteria or agar in the bottom plate. Sealed plate sets were incubated at room temperature and the radii of the fungal colonies were measured after 5 days. bVOC antifungal activity was calculated for each culture expressed as percentage growth inhibition (PGI) (Zygadlo et al., 1994), calculated as PGI (%) = 100 [(GC-GT)/GC], where GC (growth control) represents the mean diameter of fungi grown in PDA, and GT (growth treatment) represents the mean diameter of fungi exposed to bVOCs in the sandwich plate assay. All bacterial isolates were screened using three to four replicates prepared from one overnight culture of the bacterium and mycelial blocks harvested from one fungal culture plate. *C. vaccinii* isolates were assayed in more detail with three overnight cultures prepared, and each culture was tested in triplicate for fungal inhibition.

### Hydrogen Cyanide Production

*C. vaccinii* MWU205, MWU328, MWU300, the QS mutants MWU205W, MWU300W, and MWU328W, and *C. subtsugae* MWU12-2387 and MWU13-2521 were grown in 50 mL KMB broth at 26°C with aeration until early stationary phase. *C. subtsugae* was included as a negative control for hydrogen cyanide (HCN) production based on prior unpublished results. Cell densities were measured as OD_600_ for normalization between isolates and replicates. Culture supernatants (1 mL) were alkalinized by the addition of 100 μL 1N NaOH (pH ≥ 11), and HCN concentrations in solution were directly measured using a cyanide probe (Lazar Research Laboratories Inc.) attached to a pH meter (Corning Inc. Corning, NY, USA) by a modification of a previously-described method (Zlosnik and Williams, 2004). Direct measurements (mV) were converted to concentration (ppm) by comparison with a standard concentration curve (*R*^2^ > 0.99). All experiments were performed at least three times.

### Reversibility of *Phoma* sp. Growth Inhibition

To determine if the inhibition of growth by *C. vaccinii* bVOCs is reversible, the fast-growing *Phoma* sp. was grown alone or with *C. vaccinii* MWU328 or MWU328W in sandwich plates at room temperature, as described above. After 5 days of co-culture, the radii of the fungal colonies were measured and then the sandwich plates were separated from each other and the plates containing bacteria were replaced with fresh lids without bacteria or agar. Fungi were then incubated for an additional 4 days at room temperature, and the radii of the fungal colonies were measured. Growth rates during and after co-culture with bacteria were compared with negative controls that did not contain bacteria.

### Light and Transmission Electron Microscopy (TEM)

Mycelial margins of actively growing fungal cultures were excised after 120 h monoculture or co-culture in the sandwich plate format and stained with lactophenol-cotton blue to record hyphal development using light microscopy (Olympus CX41 and CellSens Entry imaging software, Hamburg, Germany). For TEM thin sectioning, hyphal apices of actively growing *Phoma* sp. in the presence or absence of *C. vaccinii* MWU328 were excised from colony margins and initially fixed for 2 h at 4°C in 0.1 M potassium phosphate buffer (pH 7.2) with 2% glutaraldehyde and post-fixed at 4°C in 1% osmium tetroxide in the same buffer for 2 h. Overnight *en bloc* staining was performed in 0.5% aqueous uranyl acetate at 4°C. Samples were dehydrated in a graded ethanol series and transitioned to propylene oxide, then infiltrated with Spurr's epoxy resin (Ann Ellis, 2018), and polymerized at 60°C for 36 h. Sections were cut to 70 nm with a Leica Ultracut-R microtome and mounted on formvar-coated copper slot grids. Grids were post-stained with 2% uranyl acetate in 50% ethanol for 8 min, followed by Sato's lead citrate (Hanaichi et al., 1986) for 4 min. Images were generated using a Philips CM12 TEM at 80 kV and acquired with a Gatan model 791 slow-scan CCD camera (1024 × 1024 pixel resolution).

### VOC Collection and Analysis by SBSE-TD-GC × GC-TOFMS

**Figure 1:**
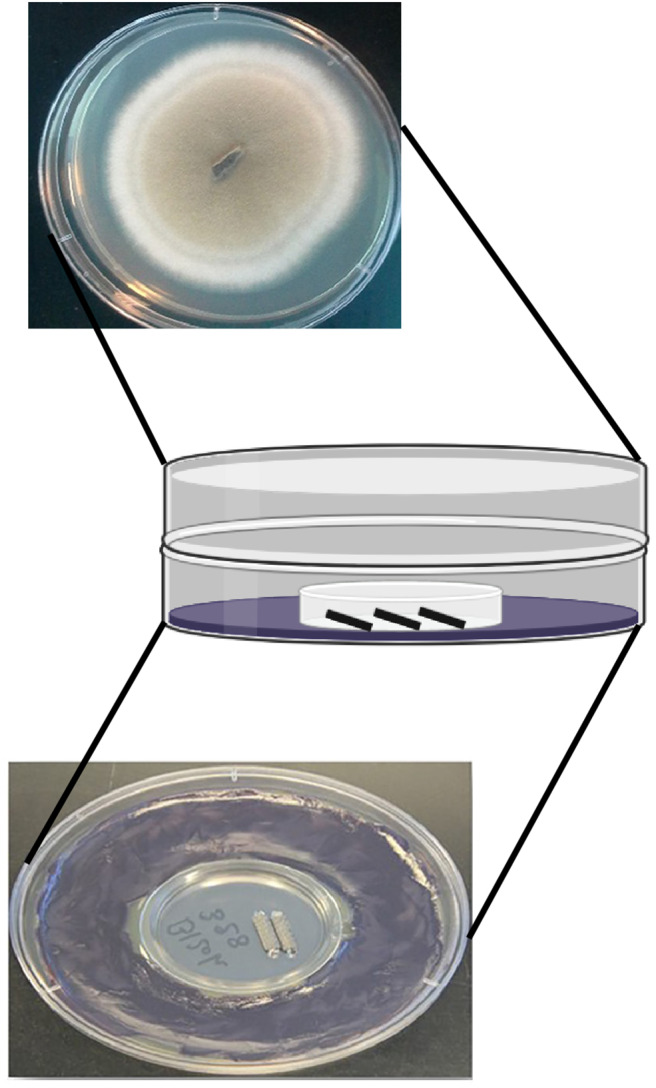
Sandwich plate device modified for sampling VOCs.

The sandwich plate was modified to capture bacterial and fungal VOCs using stir-bar sorptive extraction (SBSE; Figure 1). Molten KMB agar was poured into a 100 × 21 mm petri dish, and an empty 35 mm petri dish was aseptically embedded in the KMB agar. After cooling, bacteria were spread on the KMB around the smaller petri dish, grown for 24 h, and three polydimethylsiloxane/ethylene glycol-coated SBSE stir bars (32 μl phase volume, 10 mm length; Twister®, Gerstel, US) were aseptically placed in the smaller dish to collect VOCs in technical replicates. The lid of the bacterial plate was replaced by a “bottom” petri dish plate containing PDA inoculated in the center with a mycelial agar block (Chaurasia et al., 2005). The two plates were then sealed with petri-seal tape and incubated at room temperature for 120 h, when the stir bars were removed and placed in individual vials, and stored at 4°C prior to analysis. Each bacterial and fungal monoculture and co-culture combination was independently assayed in six biological replicates.

VOCs collected on the SBSE stir bars were analyzed via thermal desorption (TD; Gerstel MPS Robotic, Maestro software version 1.5.3.2, Linthicum Heights, MD) coupled with comprehensive two-dimensional gas chromatography—time-of-flight mass spectrometry (GC × GC-TOFMS; LECO Pegasus 4D and Agilent 7,890 GC, ChromaTOF software version 4.71, LECO Corporation, St. Joseph, MI). The stir bars were dry purged under helium at 2 mL/min for 20 min at 50°C, then desorbed at 220°C for 5 min. Desorbed VOCs were transferred to the cooled injection system with a transfer line temperature of 240°C and cryofocused onto an unpacked glass inlet liner at −80°C. VOCs were injected at 275°C for 3 min, without split. Chromatographic analysis was performed using a 2D column set consisting of an Rxi-624Sil (60 m × 250 μm × 1.4 μm (length × internal diameter × film thickness); Restek, Bellefonte, PA) as the first dimension column, and a Stabilwax (1.3 m × 250 μm × 0.5 μm; Restek) as the second dimension column. The primary oven was initiated at 35°C for 0.5 min, ramped at a rate of 5°C/min to 230°C, and held at that temperature for 5 min. The secondary oven and modulator were maintained at a +5°C and +20°C offset from the primary oven, respectively. A 2 s modulation period (alternating 0.5 s hot and cold pulses) was used with helium as the carrier gas at a flowrate of 2 mL/min. The TOFMS was operated as follows: electron impact at −70 eV; acquisition range: 35–400 *m*/*z*; acquisition rate: 100 spectra/s; and ion source temperature: 250°C. An external alkane standards mixture (C_8_-C_20_; Sigma-Aldrich, St. Louis, MO), was sampled multiple times for use in determining retention indices. The injection, chromatographic, and mass spectrometric methods for analyzing the alkane standards was as described above for SBSE sample analyses.

### Processing and Analysis of Chromatographic Data

Data collection, processing, and alignment were performed using ChromaTOF software version 4.71 with the Statistical Compare package (Leco Corp.). For peak identification, the baseline was set through the middle of the noise, and the signal-to-noise (S/N) cutoff for the initial peak finding was set to 50 for a minimum of two apexing masses. Subpeaks were combined when the mass spectral match score was ≥ 600 (out of 1,000) and the second dimension retention time shift was ≤ 100 ms for subsequent modulation periods.

Peak alignment was performed using the Statistical Compare feature of ChromaTOF. For the alignment of peaks across chromatograms, the maximum first and second dimension retention time deviations were set at 6.0 and 0.2 s, respectively, and the inter-chromatogram spectral match threshold was set at 600. A second round of peak discovery was performed using a reduced S/N of 5 to include low-abundance peaks that appeared in at least one chromatogram at S/N ≥ 50. Peaks were identified by forward and reverse searches of the NIST 2011 library. Peaks were assigned a putative identification based on mass spectral similarity and retention index (RI) data. Peaks with a level 2 or level 3 identification (Sumner et al., 2007) have ≥ 800 mass spectral match by a forward search of the NIST 2011 library. Level 2 peaks also have RIs that are consistent with the midpolar Rxi-624Sil stationary phase, as previously described (Bean et al., 2016), but using a modified RI range of 0–43%. Level 2 and 3 compounds are assigned to chemical functional groups based upon characteristic mass spectral fragmentation patterns and second dimension retention times. Level 4 compounds do not have mass spectral or RI data of sufficient quality for identification, and are reported as unknowns.

### Statistical Analyses

Differences between fungal growth inhibition from wild type vs. QS mutant isolates of *C. vaccinii* were statistically analyzed in each targeted fungus using Kruskal–Wallis and Mann–Whitney *U*-tests [Statistical Package for the Social Sciences (SPSS) 7 software] and an alpha of 0.05. All statistical analyses of metabolomics data were performed using R version 3.5.3 (R Foundation for Statistical Computing). Before statistical analyses, compounds eluting prior to 358 s (acetone retention time) and siloxanes (i.e., chromatographic artifacts) were removed from the peak table. The relative abundance of compounds across chromatograms was normalized using probabilistic quotient normalization (PQN) (Dieterle et al., 2006). Intraclass correlation coefficients (ICCs) were calculated, using R ICC package version 2.3.0, on samples with three technical replicates; peaks with an ICC <0.4 were not further processed. Analytes were retained for further analysis if the arithmetic means of sample peak areas were two-fold greater in any sample compared to the media-only controls, and significantly greater in abundance using Welch's *t*-test with an alpha of 0.05. Geometric means of the technical replicates were calculated and used for all subsequent comparisons. Principal component analysis (PCA) was performed with the biological replicates as observations and the absolute peak intensities (mean-centered and scaled to unit variance) as variables.

## Results

### Preliminary Evaluation of the Effect of VOCs on the Growth of Fungi

We used an agar-diffusion assay to prescreen 68 bacterial isolates from wild and cultivated cranberry bogs for antifungal activity, and identified 21 bacterial isolates representing 13 genera that could inhibit the growth of *Trichoderma* sp., *Phoma* sp., and *Colletotrichum* sp. (data not shown). These 21 isolates were analyzed for antifungal bacterial volatile organic compounds (bVOC) activity using a co-cultivation method that restricts microbial interactions to the gas phase. We quantified antifungal activity by measuring radial growth inhibition of bVOC-exposed *Trichoderma* sp., *Phoma* sp., *Colletotrichum* sp., *Coleophoma* sp., and *P. cinnamomi* colonies compared to unexposed controls. *Pseudomonas chlororaphis* 30–84 was used as a positive control since it is known to produce antifungal VOCs (Popova et al., 2014). The bacterial isolates varied in antifungal activity between fungi, and fungi responded to each bacterium differently (Table 1). *Trichoderma* sp., *Phoma* sp., *Colletotrichum* sp., *Coleophoma* sp., and *P. cinnamomi* exposed to bVOCs displayed mean radial growth inhibition ranging from 3 to 97%, in comparison with the no-bacteria controls. The radial growth of *Colletotrichum* sp. was inhibited by most of the bacteria, but even in the presence of *Burkholderia cepacia, Xylophilus ampelinus, Ewingella americana, Serratia marcescens*, and *Delftia* sp., which did not inhibit radial growth, the mycelium was altered such that the development of normal, high-density hyphae was restricted to near the inoculum block, and only low-density hyphae were evident beyond the inoculation site (data not shown). The greatest fungal inhibitions were a result of *Chromobacterium* spp. VOCs, particularly *C. subtsugae* MWU13-2521 and *C. vaccinii* MWU205, which inhibited *Coleophoma* sp. growth by 96 and 97%, respectively, and *Phoma* sp. by 63 and 82%, respectively. In comparison, the *P. chlororaphis* positive control inhibited the radial growth of the five tested fungi between 10 and 39% (Table 1).

**Table 1:** The percentage of growth inhibition of fungi due to exposure to bacterial VOCs vs. the growth control (i.e., negative control).

	**Percentage of fungal growth inhibition**				
**Bacterial species and strains**	***Trichoderma* sp**.	***Phoma* sp**.	***Colletotrichum* sp**.	***Coleophoma* sp**.	***Phytophthora cinnamomi***
*Pseudomonas chlororaphis* 30–84	23 ± 8	39 ± 8	10 ± 1	39 ± 3	27 ± 4
*Bacillus thuringiensis* MWU12-2420	3 ± 3	25 ± 6	4 ± 5	33 ± 6	29 ± 2
*Bacillus cereus* MWU14-2326	8 ± 6	20 ± 2	14 ± 10	18 ± 11	7 ± 1
*Aquitalea* sp. MWU14-241	15 ± 12	36 ± 10	4 ± 9	42 ± 3	36 ± 4
*Pseudomonas* sp. MWU13-2590	34 ± 19	71 ± 4	9 ± 3	59 ± 4	28 ± 4
*Pseudomonas* sp. MWU12-2517	14 ± 5	23 ± 2	20 ± 14	31 ± 5	39 ± 3
*Pseudomonas* sp. MWU15-20650	21 ± 4	57 ± 5	24 ± 6	42 ± 2	20 ± 7
*Burkholderia cepacia* MWU13-2092	18 ± 4	14 ± 7	−2 ± 7	45 ± 6	34 ± 3
*Burkholderia tropica* MWU12-2056	34 ± 19	33 ± 5	31 ± 8	52 ± 6	50 ± 3
*Xylophilus ampelinus* MWU14-20187	21 ± 7	39 ± 6	−16 ± 2	49 ± 22	14 ± 2
*Acinetobacter calcoaceticus* MWU13-2536	9 ± 8	46 ± 12	4 ± 19	59 ± 2	22 ± 5
*Ewingella americana* MWU14-20116	6 ± 4	30 ± 5	−2 ± 11	50 ± 12	35 ± 4
*Serratia marcescens* MWU13-2543	9 ± 3	35 ± 8	−1 ± 8	35 ± 6	23 ± 3
*Lysinibacillus* sp. MWU14-2414	25 ± 9	16 ± 5	4 ± 7	41 ± 2	19 ± 4
*Paenibacillus* sp. MWU13-2602	3 ± 5	15 ± 2	7 ± 13	33 ± 3	14 ± 2
*Enterobacter* sp. MWU13-2507	11 ± 7	28 ± 6	10 ± 8	48 ± 3	30 ± 0
*Delftia* sp. MWU13-3324	13 ± 2	32 ± 10	−13 ± 4	69 ± 1	22 ± 1
*Chromobacterium subtsugae* MWU13-2521	21 ± 8	63 ± 9	7 ± 1	96 ± 4	5 ± 3
*Chromobacterium vaccinii* MWU205	23 ± 5	82 ± 4	20 ± 10	97 ± 3	18 ± 1
*Chromobacterium vaccinii* MWU300	16 ± 4	81 ± 2	10 ± 3	77 ± 4	26 ± 4
*Chromobacterium vaccinii* MWU328	16 ± 5	81 ± 3	25 ± 6	92 ± 4	27 ± 0

*Values represent mean ± standard error of the mean from three to four replicates*.

### Antifungal Activity of *C. vaccinii* VOCs

**Figure 2:**
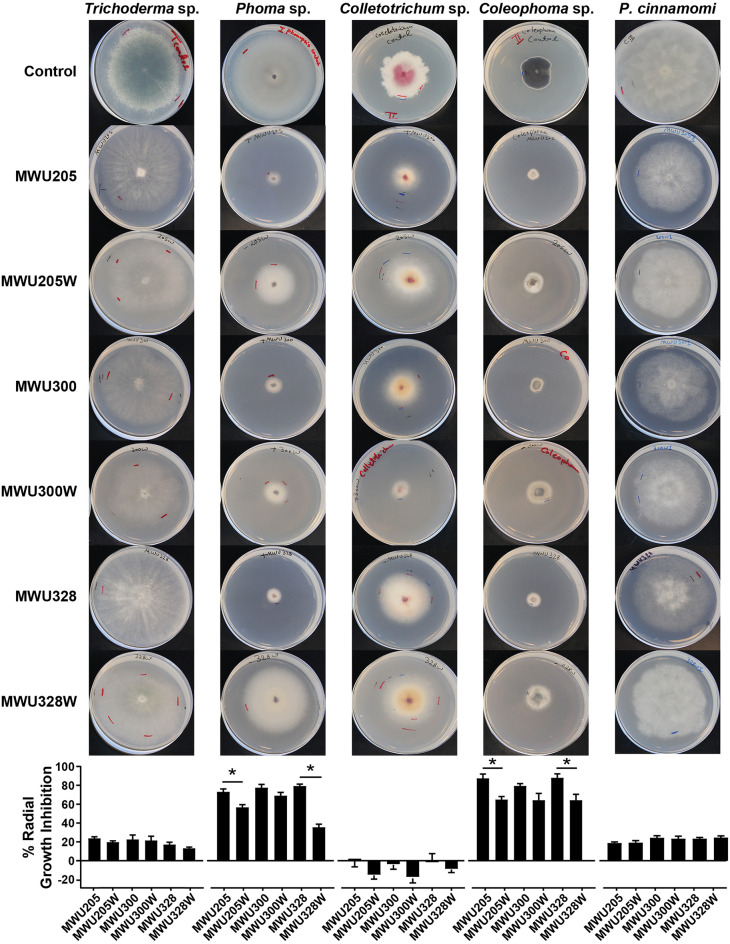
Morphological and fungistatic effects of bVOCs produced by *Chromobacterium vaccinii*. Wild-type isolates MWU205, MWU300, and MWU328, and quorum sensing mutants MWU205W, MWU300W, and MWU328W were assayed for activity of bVOCs against four fungal isolates from cranberry bogs, *Trichoderma* sp., *Phoma* sp., *Colletotrichum* sp., and *Coleophoma* sp., and the oomycete *Phytophthora cinnamomi*. Error bars indicate standard error of the mean (SEM). *p ≤ 0.05.

Due to its high levels of fungal inhibition and broad activity against the fungi and oomycete we tested, we selected the recently characterized Gram-negative bog soil β-proteobacterium *C. vaccinii* (Soby et al., 2013) for further study. This species is notable for its deep purple pigmented phenotype (Soby et al., 2013) and for its ability to kill *Aedes* sp. mosquito larvae *in vitro* (Martin and Soby, 2016), with both phenotypes regulated by quorum sensing (QS). We assayed fungal growth inhibition by the VOCs of *C. vaccinii* wild-type isolates MWU205, MWU300, and MWU328 and their QS-deficient (CviR^−^) mutants MWU205W, MWU300W, and MWU328W against *Trichoderma* sp., *Phoma* sp., *Colletotrichum* sp., *Coleophoma* sp., and *P. cinnamomi* (Figure 2). Radial growth inhibition in the presence of wild-type *C. vaccinii* MWU205, MWU300, and MWU328 was most pronounced in the fruit pathogens *Phoma* sp. (74–79%) and *Coleophoma* sp. (80–88%; Figure 2). The foliar pathogen *Colletotrichum* sp. was not inhibited by these bacteria as assayed by radial growth, and *Trichoderma* sp. and *P. cinnamomi*, two species normally found in the soil, were inhibited by a much smaller amount (13–43%). The QS mutants reduced radial growth of *Phoma* sp. and *Coleophoma* sp., but far less than their cognate wild-type strains, with significant differences in all combinations except for the MWU300 and MWU300W pair (Figure 2). There were no significant differences in radial growth between incubation with wild type and QS mutant strains in *Trichoderma* sp., *Colletotrichum* sp., or *P. cinnamomi*, likely because of the small amount of inhibition associated with wild type bVOC production.

**Figure 3:**
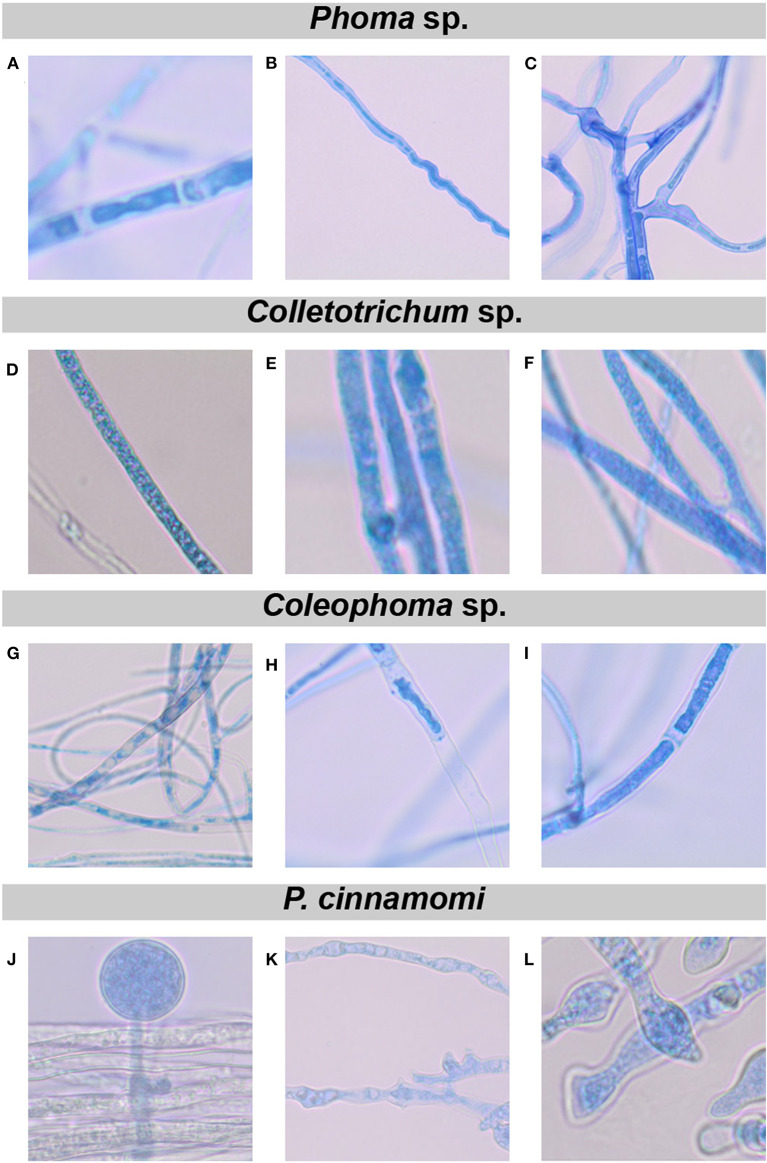
Morphological changes in *Phoma* sp. **(A–C)**, *Colletotrichum* sp. **(D–F)**, *Coleophoma* sp. **(G–I)** and *Phytophthora cinnamomi* **(J–L)**, by bVOCs produced by *C. vaccinii* MWU328. The column ADGJ represents untreated hyphae for each of the fungi. Columns BEHK and CFIL show hyphae from each of the fungi after exposure to bVOCs. **(B,D,G,K)** Are shown at 40x magnification, and **(A,C,E,F,H,I,J, L)** are shown at 100x.

*C. vaccinii* bVOCs also affected the colony gross morphology of the exposed fungi. For example, *Trichoderma* sp., *Colletotrichum* sp., and *Coleophoma* sp. showed a reduction in pigmentation in the presence of bVOCs (Figure 2). Although we did not assign pigmentation differences to specific cell types, some of the loss of pigment may have been due to reduction in sporulation, but some was clearly associated with mycelium. For example, *Colletotrichum* sp. strain MWU-UMCS9301 does not sporulate well on PDA, but there was a visible reduction in pigmentation on that medium. The radial growth of *Trichoderma* sp., *Colletotrichum* sp., and *P. cinnamomi* was not significantly inhibited, and in some cases slightly promoted, but mycelium density was noticeably reduced (Figure 2), suggesting that bVOCs had an effect that resulted in hyphal tip hypertrophy at the expense of normal development. Structural deformations have previously been observed in phytopathogenic fungi exposed to bVOCs (Moore-Landecker and Stotzky, 1973; Chaurasia et al., 2005; Giorgio et al., 2015). In order to evaluate the effect of bVOCs on fungal cellular structures, light microscopy was performed on hyphae of *Phoma* sp., *Colletotrichum* sp., *Coleophoma* sp., and *P. cinnamomi* after exposure to *C. vaccinii* MWU328 bVOCs (Figure 3). We selected MWU328 for this analysis because it demonstrated antifungal activity against all fungi and oomycete tested, but not complete growth inhibition. In *Coleophoma* sp., hyphal cell membranes had pulled away from the cell wall, and there was a higher than normal proportion of empty cells in treated (Figures 3H,I) vs. control hyphae (Figure 3G). There was abnormal swelling and deformation of hyphae in *Phoma* sp., *Colletotrichum* sp., and *P. cinnamomi* (Figures 3B,C,E,F,K,L), as well as an absence of sporangia in *P. cinnamomi*.

### VOC Interactions Between *C. vaccinii* and *Phoma* sp.

To examine the effects of antifungal bVOC exposure in greater detail, we selected *C. vaccinii* MWU328, *C. vaccinii* MWU328W, and *Phoma* sp. MWU-UMCS9302 for additional analyses. We selected these isolates because *Phoma* sp. is fast growing, and the wild-type *C. vaccinii* strain MWU328 did not fully inhibit its growth, which facilitated investigations of fungal morphology, fungal VOCs (fVOCs), and the reversibility of growth inhibition when *C. vaccinii* VOCs are removed.

#### *Phoma* sp. Cellular Structural Abnormalities Caused by *C. vaccinii* bVOCs

**Figure 4:**
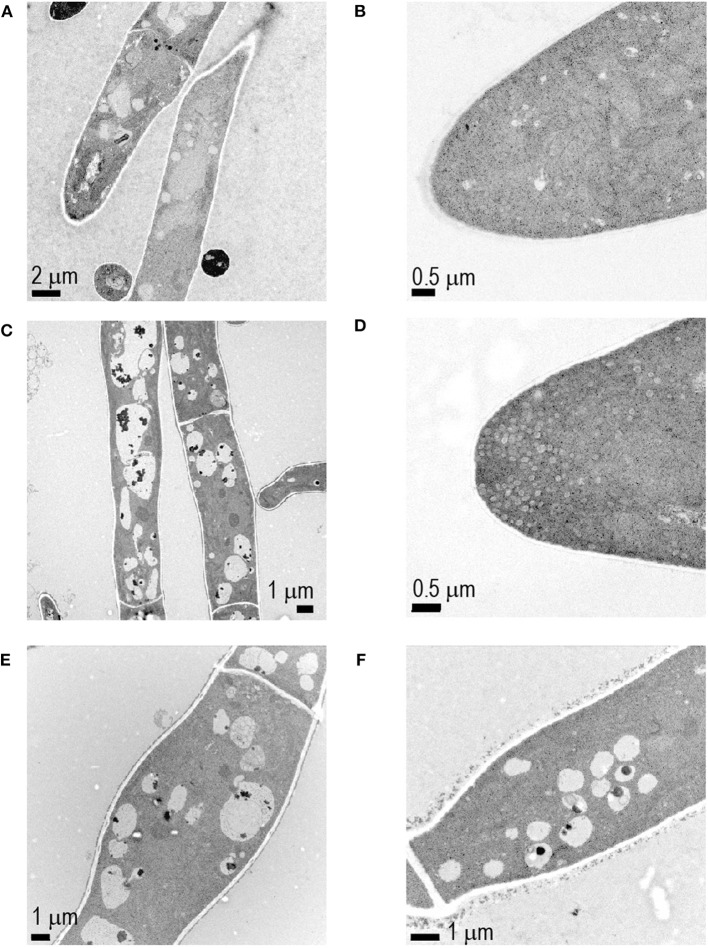
Ultrastructural analysis of *Phoma* sp. mycelia exposed to the bVOCs of *C. vaccinii* MWU328 **(C–F)**. *Phoma* sp. mycelia not exposed to bVOCs were used as controls **(A,B)**.

*Phoma* sp. either exposed or unexposed to *C. vaccinii* MWU328 bVOCs was examined by transmission electron microscopy (Figure 4). The cytoplasm of bVOC-exposed cells was highly vacuolated with the inclusion of large dark-staining bodies (Figures 4C,E,F), and the presence of abnormally large and disorganized Spitzenkörper (Figure 4D), which are not apparent in untreated control hyphal tips (Figures 4A,B). Treated mycelia were also characterized by the swelling of individual cells (Figures 4E,F), and a dark-staining material of unknown composition associated with the external surface of cell walls (Figure 4F). Untreated controls had an average cell wall thickness of 1.04 mm (SD = 0.23), and treated mycelia had an average cell wall thickness of 1.50 mm (SD = 0.49; *p* = 0.0002).

#### Reversibility of *Phoma* sp. Growth Inhibition by *C. vaccinii* VOCs

To determine if the inhibition of growth by *C. vaccinii* bVOCs is reversible, we measured the growth of *Phoma* sp. during and after exposure to MWU328 and MWU328W bVOCs in the sandwich plate assay. *Phoma* sp. growth rates were significantly different after 5 days exposure to *C. vaccinii* bVOCs compared to the control (*p* = 3 × 10^−10^). *Phoma* sp. grew at an average rate of 0.5 mm/day in the control compared to 0.1 mm/day in the presence of wild type MWU328, and 0.4 mm/day in the presence of QS mutant MWU328W. After the bacteria cultures and control blank agar were replaced with empty petri plate lids on day five, *Phoma* sp. that had been previously exposed to MWU328 and MWU328W bVOCs grew at the same rate as the control (MWU328 vs. control, *p* = 0.083; MWU328W vs control, *p* = 0.074), showing that the growth inhibition is reversible at the bVOC concentrations produced in these experiments.

#### Characterizing the VOCs of *C. vaccinii* and *Phoma* sp. by SBSE-GC × GC-TOFMS

Stir bar sorptive extraction and comprehensive two-dimensional gas chromatography—time-of-flight mass spectrometry (SBSE-GC × GC-TOFMS) was used to collect and analyze the VOCs produced by *C. vaccinii* strain MWU328 and its less-fungistatic QS mutant MWU328W in the presence or absence of *Phoma* sp. MWU-UMCS9302. The VOCs of *Phoma* sp. in monoculture and of media-only controls were also analyzed by SBSE-GC × GC-TOFMS (Supplementary Figure 2). After data alignment and removal of chromatographic artifacts, 1,931 non-redundant peaks were detected across the 84 samples, which included six biological replicates of each experimental combination. The peak list was then filtered to include only compounds that were detected in reproducible intensities across biological replicates and present in the biological samples in at least two-fold greater concentration relative to media controls. This step removed 1,878 peaks. The remaining 53 peaks were relatively evenly distributed across the five microbial samples (Table 2, Supplementary Table 2). Based upon mass spectral and chromatographic data, we were able to assign putative identities to eight VOCs, which included known bioactive compounds such as dimethyl disulfide, dimethyl trisulfide, indole, 1-octanol, and octanoic acid (Kai et al., 2009; Schulz et al., 2010; Forlani et al., 2011; Groenhagen et al., 2013; Giorgio et al., 2015; Lo Cantore et al., 2015). For the unnamed compounds, we were able to assign chemical classifications to 30 of them. The remaining 15 VOCs are classified as unknowns, due to a lack of mass spectral or chromatographic data of sufficient quality for putative identification.

**Table 2:** Volatile metabolites that are significantly more abundant than blank media as a function of sample type.

**Compound ID**	***Cv***	***P-Cv***	***P***	***P-Cv*W**	***Cv*W**
Thiazole				**↓-**	
Disulfide, dimethyl		**-↑**		**↑-**	
UNK-3		**-↓**		**↓-**	
CA-4					
CA-5		**-↓**			
UNK-6		**-↓**			
HC-7					
HC-8					
OTH-9					
1,2-Ethanediol, diacetate		**-↓**			
S,N-11				**↓-**	
Dimethyl trisulfide		**↓-**		**↑-**	
N-13					
CA-14					
N-15					
EST-16		**-↓**			
1-Octanol		**↓-**			
CA-18					
S-19					
UNK-20					
UNK-21					
UNK-22					
ETH-23					
Octanoic acid					
CA-25		**-↓**			
HC-26					
UNK-27					
UNK-28		**↓↓**		**↓-**	
ETH-29		**-↓**			
ETH-30		**-↓**		**↓-**	
ETH-31					
Indole					
EST-33		**↑-**		**-↑**	
EST-34					
UNK-35					
UNK-36					
UNK-37		**↑↑**			
EST-38		**-↓**		**↓-**	
ETH-39					
T-40					
UNK-41		**-↓**			
N-42					
UNK-43					
ARO-44					
ARO-45		**-↓**		**↓-**	
α-Calacorene					
UNK-47					
EST-48		**↑-**		**-↑**	
UNK-49		**↑-**		**↓-**	
ETH-50					
ARO-51					
UNK-52		**↑-**		**-↑**	
N-53					
			*C. vaccinii* MWU328 monocultures (*Cv*)		
			*Phoma* sp., MWU328 co-cultures (*P*-*Cv*)		
			*Phoma* sp. monocultures (*P*)		
			*Phoma* sp., MWU328W co-cultures (*P*-*Cv*W)		
			*C. vaccinii* MWU328W monocultures (*Cv*W)		

*The numbers of replicates the VOCs were detected in are indicated by the color saturation. In co-cultures, the hypothesized source of the VOCs is indicated by color, and arrows indicate which peak intensity significantly (p <0.05) increased (↑) or decreased (↓) in co-culture compared to the two constituent monocultures; a dash (-) indicates no change. Functional classes include: ARO, aromatic hydrocarbon, CA, carboxylic acid, EST, ester, ETH, ether, HC, hydrocarbon, N, nitrogen containing, OTH, other, S, sulfur containing, T, terpene, UNK, unknown. Additional compound information and the peak intensities measured in each experimental condition are provided in Supplementary Table 2*.

Among the 53 microbially-derived VOCs, 14 were uniquely produced by *Phoma* sp. (i.e., detected in a majority of *Phoma* sp. monocultures and one or zero MWU328 or MWU328W replicates), including several carboxylic acids and aromatic hydrocarbons (Table 2). Eight VOCs were detected that were unique to the bacteria, including indole (Yu et al., 2000; Blom et al., 2011; Lazazzara et al., 2017), which has been previously reported as a bVOC. Among the bVOCs, there were two compounds that were only detected in the wild-type MWU328, CA-18 and UNK-27. Two compounds, N-13 and indole, were solely produced by the QS mutant MWU328W. The detection of indole in the headspace of the unpigmented strain MWU328W is consistent with its *cviR* mutation, because violacein, the quorum-regulated purple pigment produced by *C. vaccinii*, is synthesized from two molecules of tryptophan (Momen et al., 1998; Rettori and Duran, 1998). The tryptophan biosynthesis operon is constitutively expressed in violacein-producing species of *Chromobacterium* at a high level to supply violacein precursors (Antonio and Creczynski-Pasa, 2004). Therefore, down regulation of the violacein synthesis pathway caused by the *cviR* mutation likely results in excess tryptophan, and thus an increase in its catabolism with the indole side chain released as a byproduct.

To filter the peak list down to potentially biogenic compounds, we took a biologically agnostic approach so as not to restrict our analyses to previously known microbial VOCs; rather we used statistical criteria to determine the list of putatively biogenic compounds by comparing VOC peak intensities in microbial cultures vs. media controls. Aside from the exclusion of siloxanes, we also took a chemically-agnostic approach, and did not exclude *a priori* compounds such as ethers, which can be generated abiologically via thermal decomposition of the polymers coating the SBSE devices. However, the list of 53 microbial VOCs includes possible analytical artifacts such as a potential phthalate (EST-33) and compounds resembling polyethylene glycol decomposition products (ETH-29, ETH-30, and ETH-39). Unlike typical chemical artifacts, these compounds statistically and reproducibly differed in intensity in the bacterial and fungal samples vs. media controls, and between one experimental biological condition and another. For example, EST-33 was present in higher concentrations in the bacterial-fungal co-cultures than in any of the monocultures, and ETH-39 was specific to cultures containing bacteria. While it is possible that the sources of these compounds are the materials that were used to culture the microbes or to collect the VOCs, our data indicate that there is a biological contribution to their production, such as the microbial degradation of petri dish materials, or the chemical reaction of biological VOCs with the sorbents used to collect them. However, additional analyses (below) indicate that these compounds are not highly relevant to the fungal inhibition and growth phenotypes we observed.

**Figure 5:**
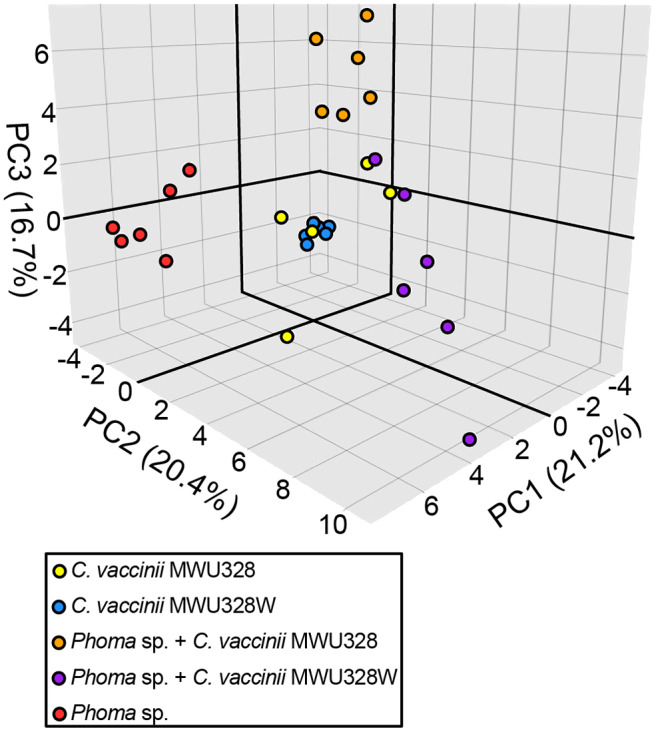
Principal component analysis score plot of monocultures of *Phoma* sp. (red), *C. vaccinii* MWU328 (Yellow), and *C. vaccinii* QS mutant MWU328W (Blue), and co-cultures of *Phoma* sp. and MWU328 (Orange) and *Phoma* sp. and MWU328W (Purple), based upon 53 biogenic VOCs. Six biological replicates were performed for each experiment.

We used principal components analysis (PCA) for an untargeted approach to evaluate the relationships between the VOCs produced by *Phoma* sp., *C. vaccinii* MWU328, and MWU328W in mono- and co-culture (Figure 5, Supplementary Figures 3, 4). Using the 53 microbial VOCs as the variables and the six biological replicates of each culture condition as the observations, the presence or absence of bVOCs vs. fVOCs explains the largest proportion of variance in the model (PC1, 21.2%). Monocultures of MWU328 and MWU328W cluster together along PC1 <0, and away from the *Phoma* sp. monocultures clustered in PC1 > 0. Importantly, the co-cultures do not fall between the fungal and bacterial monoculture clusters, demonstrating that the VOCs of the co-culture are not a linear combination of fVOCs and bVOCs (Figure 5, Supplementary Figure 3). Further, the two co-cultures do not cluster together as the two monocultures of bacteria do, showing that the volatile metabolomes created by co-culture differ based on the presence or absence of intact *C. vaccinii* quorum sensing. PCA biplots reiterate many of the observations we made in the analysis of the VOC peak tables (Supplementary Figure 4). For example, most of the VOCs that are only produced by *Phoma* sp. show strong loadings on PC1 > 0. However, the loadings also reveal additional VOCs that are associated with more than one culture condition. For example, the loadings in PC1 vs. PC3 (Supplementary Figure 4) indicate that three aromatic hydrocarbons—ARO-45, ARO-44, and ARO-51–are detected in *Phoma* sp. monocultures as well as in *Phoma* sp. co-culture with MWU328.

The induction or suppression of monoculture VOCs in the co-cultures are evidence of VOC-mediated interactions between *C. vaccinii* and *Phoma* sp. For example, in both co-cultures there was a statistically significant increase in the production of UNK-52, a VOC produced in low abundance and low frequency in the bacterial monocultures, but in very high concentrations in the co-cultures (Table 2 and Supplementary Table 2). *Phoma* VOCs HC-26 and HC-7 increased in concentration more than 10-fold (though not significantly) and α-calacorene was detected with higher frequency in the presence of wild-type *C. vaccinii*, in spite of suppressed fungal growth. We also observed the inhibition of many of the *Phoma* sp. VOCs in one or both co-cultures, and the inhibition of wild type and/or mutant bVOCs, such as N-42 and T-40. Some of the most interesting interactions, however, are characterized by VOCs that are not observed in any of the three monocultures (or are observed in very low abundance and frequency), but are robustly detected in the co-cultures (e.g., S-19, ETH-31, UNK-36, and UNK-37), or VOCs detected in both bacterial and fungal monocultures but significantly reduced in co-culture (e.g., UNK-28), suggesting feedback loops between the bacterium and fungus mediated by volatile metabolites. Overall, amongst the 106 possible observations of VOCs in co-cultures (53 VOCs × 2 co-cultures) there were 33 statistically significant changes in VOC concentration compared to monocultures, affirming that both the bacterium and the fungus are altering substantial proportions of their volatile metabolomes in response to one another.

#### Putative Inhibitory VOCs

To determine which *C. vaccinii* VOCs may be inhibiting *Phoma* sp. growth, we took two main approaches. The first approach was to distinguish putative inhibitory compounds that are consistently produced by wild type MWU328 in our experimental system in greater abundance than the QS mutant MWU328W in monoculture, and that are detectable in the MWU328 co-cultures with *Phoma* sp. The second approach was to evaluate potentially inhibitory VOCs that are absent or produced in low abundance in the MWU328 monoculture, but produced in higher abundance by the bacterium when it detects the presence of *Phoma* sp. VOCs. We refer to these two sets of VOCs as the consistent and inducible VOCs, respectively.

For inducible VOCs to be considered as candidates for the QS-regulated antifungal VOCs, they should not be detected in the fungus monocultures, they should be detected in higher frequency and/or concentration in wild-type bacteria co-culture vs. the QS mutant co-culture, and they must be absent or detected in low frequency or concentration in MWU328 monoculture vs. co-culture with the fungus. However, because there was some limited inhibition of the fungus by MWU328W, it is possible that the putative fungistatic VOCs are detectable in co-cultures with mutant bacteria, in which case they should be present in substantively lower concentrations than in wild type co-cultures. Of the bVOCs that were induced by the presence of *Phoma* sp., none fit these criteria. Therefore, we postulate that the QS-regulated fungistatic VOCs are consistently produced by the wild-type bacterium under the conditions we tested.

**Figure 6:**
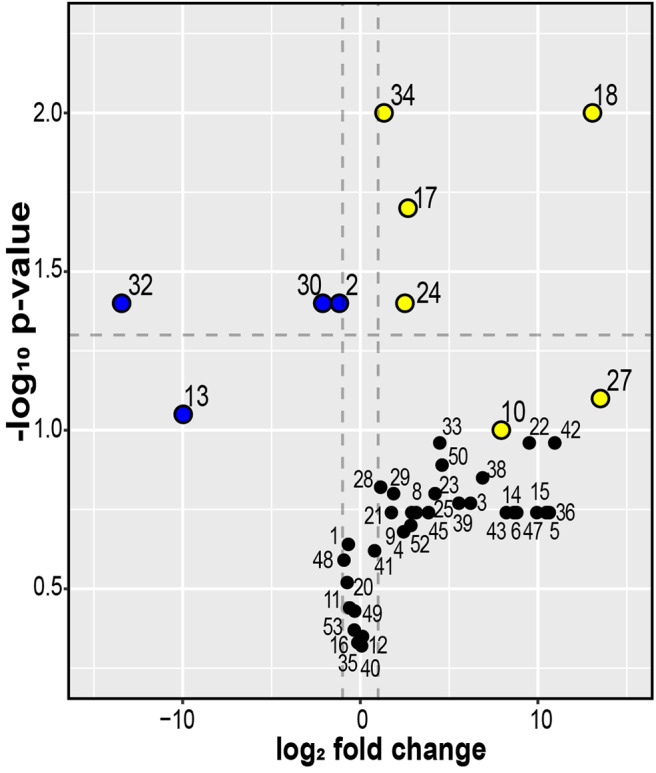
Volcano plot of the log_2_ fold-change difference in VOC abundance for MWU328 relative to MWU328W in monocultures. Dashed lines indicate *p* < 0.05 and log_2_ fold change > 1 or < −1. Metabolites in blue or yellow are more abundant in the mutant or wild type, respectively (*p* < 0.1). The fold-changes and *p*-values are provided in Supplementary Table 3. Compound IDs: 2 = dimethyl disulfide; 10 = 1,2-Ethanediol, diacetate; 13 = N-13; 17 = 1-Octanol; 18 = CA-18; 24 = Octanoic acid; 27 = UNK-27; 30 = ETH-30; 32 = Indole; 34 = EST-34.

The qualitative and quantitative data indicate that wild-type *C. vaccinii* MWU328 produces more VOCs at higher concentrations in monoculture than its QS mutant. Of the 45 VOCs detected in MWU328 or MWU328W in monoculture, 30 were detected in at least two-fold higher concentration in the wild type vs. the quorum-insensitive mutant (Figure 6, Supplementary Table 3). Of these, four compounds were significantly greater in wild type (*p* < 0.05), and compounds CA-18 and UNK-27 were not detectable in the QS mutant. For compounds to be candidates as fungistatic VOCs, they must be detectable in co-culture, and not produced by the fungus [i.e., not detected in *Phoma* sp. monocultures, or detected in the same, low concentrations in *Phoma* sp. monocultures and the media blanks (Supplementary Table 2)]. 1-Octanol, CA-18, and UNK-27 fit these additional criteria, making them candidate inhibitory VOCs that are consistently produced under QS regulation by *C. vaccinii*.

To address whether hydrogen cyanide, which is undetectable in our GC × GC-TOFMS analysis, is responsible for the fungal inhibition by *C. vaccinii* VOCs, we quantified the amount of HCN produced by *C. vaccinii* in KMB broth (Table 3). HCN concentration at early stationary phase ranged from 54 to 63 ppm for *C. vaccinii* wild-type isolates. The QS mutants also produce HCN, but at far lower concentrations. Under the same conditions, HCN concentrations ranged from 21 to 34 ppm. The negative controls *C. subtsugae* MWU12-2387 and MWU13-2521 produced 6 ppm and 9 ppm, respectively, which is marginally above the signal-to-noise threshold for the assay, yet this species demonstrated strong inhibition of several of the fungi we tested (Table 1). Therefore, in addition to the three putative inhibitory *C. vaccinii* VOCs detected by GC × GC-TOFMS, HCN can be contributing to the QS-regulated fungal inhibition we observed, but is unlikely to be wholly responsible.

**Table 3:** Concentrations of hydrogen cyanide produced by *C. vaccinii* and *C. subtsugae* grown in KMB to early stationary phase.

***Chromobacterium* isolate**	**HCN concentration (ppm)**	**Number of replicates**
*C. vaccinii* MWU205	54.4 ± 1.8	108
*C. vaccinii* MWU300	58.5 ± 1.9	36
*C. vaccinii* MWU328	63.3 ± 2.9	36
*C. vaccinii* MWU205W	23.7 ± 2.3	108
*C. vaccinii* MWU300W	20.5 ± 1.7	36
*C. vaccinii* MWU328W	33.5 ± 3.2	36
*C. subtsugae* MWU12-2387	6.4 ± 2.4	4
*C. subtsugae* MWU13-2521	8.9 ± 0.03	3

*Values represent the mean ± standard error of the mean*.

## Discussion

Twenty one of 68 bacterial isolates we pre-screened from wild and cultivated cranberry bogs had significant antifungal bVOC activity against *Trichoderma* sp., several important plant pathogenic fungi, and the oomycete *P. cinnamomi*, indicating that VOC-mediated semiotic interactions between kingdoms is fairly widespread among soil bacteria. The effects on fungi were wide-ranging, and they reflected some degree of pairwise specificity in bacterial-fungal interactions. Even when growth inhibition was not observed, changes in gross fungal colony morphology, such as reduced sporulation, pigmentation, and hyphal density were common. The combination of fungal growth inhibition and morphology changes we observed demonstrate that there is broad biological activity across the bacterial volatile metabolome.

The strongest fungal growth inhibition was produced by *C. vaccinii* bVOCs against *Phoma* sp. and *Coleophoma* sp. Because *Phoma* sp. was not completely inhibited by *C. vaccinii*, we could use this pairing as a model to characterize the cellular impacts of antifungal bVOCs. We observed cytological abnormalities such as thickened cell walls, increased vacuolization, intracellular walls with occasional breakage or incomplete development, electron-dense inclusions within the cytoplasm and vacuoles, the presence of disorganized Spitzenkörper, and the accumulation of an unknown extracellular electron-dense material indicating that the fungus is under severe physiological stress. The fact that no mitochondrial abnormalities occur in *Phoma* sp. may offer a clue as to what other cellular processes are affected by the bVOCs, but are likely unrelated to cellular energy production via the electron transport chain. We observed that once the *C. vaccinii* bVOCs are removed, the growth rate of *Phoma* sp. is the same rate as the untreated control, suggesting that there is not long-term damage to the fungus. However, the disorganized Spitzenkörper and other cytological changes in the presence of bVOCs suggest that recovery from growth inhibition may not be as simple as resuming mitosis and cell elongation, and recovery may be a dose-dependent phenomenon, which we have not yet tested. The dose effects and the mechanism (or mechanisms) involved in fungal growth inhibition by *C. vaccinii* bVOC are the subjects of ongoing work.

The involvement of quorum sensing in the regulation of some component of the VOC suite produced by *C. vaccinii* suggests that the set of VOCs produced at any given time is influenced by the relative population density of members of the same species, of other species that produce the same quorum sensing autoinducer, or the presence of quorum quenching fVOCs. Based on our comparative analyses of the VOCs from mono- and co-cultures with *C. vaccinii* MWU328 and MWU328W, we identified four candidate fungistatic VOCs, 1-octanol, CA-18, UNK-27, and HCN, which we presume to be under quorum regulation. More than a dozen alcohols and carboxylic acids have been previously identified as fungistatic or fungicidal (Giorgio et al., 2015; Alijani et al., 2019; Osaki et al., 2019), lending precedence to the putative antifungal VOCs 1-octanol and CA-18, though the link between quorum regulation and antifungal alcohol or carboxylic acid bVOCs has not been established. However, the quorum regulation of antifungal VOCs has been previously demonstrated with acetophenone and 2-aminoacetophenone, quorum-sensing-related molecules produced by *Pseudomonas* sp. (Kesarwani et al., 2011), which have been shown to significantly inhibit *Phytophthora infestans* direct sporangial germination (De Vrieze et al., 2015).

Additional known fungistatic VOCs were detected in our analyses, such as dimethyl trisulfide (Barbieri et al., 2005; Fernando et al., 2005; Kai et al., 2009; Groenhagen et al., 2013; De Vrieze et al., 2015; Giorgio et al., 2015) and octanoic acid (Schulz et al., 2010), but they were not differentially expressed between MWU328 and its QS mutant, and therefore were not identified as the QS-regulated fungistatic VOCs in our experiments. We note that MWU328W mildly inhibited *Phoma* sp. growth, which may have been due to one, some, or all of these known bioactive VOCs. Additionally, a third to a half of HCN production is maintained in the QS mutants, so some of the residual fungal growth inhibition observed in these mutants may be due to HCN. However, *C. subtsugae* does not produce appreciable amounts of HCN, yet it has good growth inhibition properties (Table 1). Therefore, we cannot attribute all of the *Chromobacterium* sp. inhibition to HCN alone. It has long been known that microbial VOCs are produced in combinations, the sum of which are responsible for antifungal activity (Moore-Landecker and Stotzky, 1973, 1974). Although we have attempted to identify individual fungistatic compounds, it should be understood that it is likely a suite of VOCs that is responsible for the gross morphological and growth inhibition effects we observed. Determining the degree to which the four candidate fungistatic VOCs can inhibit growth independently, in combination, or in complex with the many other VOCs produced by *C. vaccinii* will require positive identification of the QS-regulated bVOCs via authentic standards, followed by bioassays against *Phoma* sp. and other fungi. These experiments are the subjects of ongoing work.

The variation in growth inhibition, and in phenotypic and morphological changes among fungi we tested suggests varying levels of fungal susceptibility to the types and concentrations of bVOCs produced. The soil and rhizosphere genera *Chromobacterium* and *Pseudomonas* were particularly effective at inhibiting fungal growth, and both of these genera include species with biocontrol activities against insects, fungi, and oomycetes (Siddiqui and Shaukat, 2004; Durán et al., 2007, 2016; Martin et al., 2007; De Vrieze et al., 2015; Hunziker et al., 2015; Ossowicki et al., 2017; David et al., 2018). The only species we tested from both wild and cultivated bogs was *C. vaccinii*, but the numbers of isolates were insufficient to test hypotheses related to antifungal VOCs, cultivation practices, and fungal disease burden. However, we noted that all three *C. vaccinii* wild-type isolates MWU205, MWU300, and MWU328 were able to limit the growth of *Phoma* sp. and *Coleophoma* sp.—two fruit rot pathogens—to <3% of the control growth rate. In contrast, *Trichoderma* sp. and *P. cinnamomi*, two species normally found in the soil where *Chromobacterium* sp. dwell, were far less susceptible to bVOC-induced inhibition for reasons that are as yet unknown. The observation that fungi isolated from, and primarily associated with the above-ground parts of the plant are more sensitive to stasis and morphological changes induced by exposure to bVOCs raises the possibility that there has been co-evolution of the soil fungi with bacteria that occupy the same niches, resulting in fungal resistance to the effects of the bVOCs. We think this hypothesis is worthy of testing and may lead to a broader understanding of semiochemical interactions in natural and engineered microbial communities.

A significant and unexpected finding from our VOC analyses is that the volatile metabolomes of both the fungus and the bacteria are altered in co-culture compared to their respective monocultures. We measured statistically significant increases in concentration of three bVOCs and an increase in detection rates of three fVOCs in co-culture compared to monoculture. Three additional VOCs were robustly detected only in co-cultures and 17 bVOCs and/or fVOCs were significantly inhibited in co-culture vs. monoculture. Intriguingly, we detected co-culture-induced fVOCs in spite of fungal growth inhibition, while also measuring statistically significant decreases in the antifungal bVOCs dimethyl trisulfide and 1-octanol, which together suggests that *Phoma* sp. produces defensive VOCs to reduce the production of antifungals by *C. vaccinii*. To our knowledge only two other studies have reported similar VOC-mediated inductions of microbial volatile metabolites. Azzollini and colleagues identified sixteen fVOCs that were induced when two wood-decaying fungi exchanged VOCs, and among them was 2-nonanone, an antifungal VOC that could suppress the growth of both fungi (Azzollini et al., 2018). Schmidt and colleagues showed that exposure to *Fusarium culmorum* VOCs could induce the production of a terpene in *Serratia plymuthica* (Schmidt et al., 2017). Our data provide an example of volatile metabolites shaping inter-kingdom microbial interactions via bVOC-fVOC feedback loops, resulting in a co-culture volatile metabolome with emergent properties.

In these experiments we have characterized the interactions between single bacterial isolates with single fungi growing in pure cultures. The reality in the soil and rhizosphere is that there are many hundreds of types of microbes present, and the simplicity of our model test system limits its direct translatability. However, based on our preliminary assays, many genera of cultivable bacteria from those environments produce fungal inhibitory bVOCs, and in the case of *Chromobacterium*, some are under quorum regulation. Thus, the soil and nearby rhizosphere are likely to be rich, dynamic, and highly biologically active signaling environments. The overall effect of bVOCs produced by soil microbes on plants, nematodes, insect larvae, and other microbes is likely to be complex, particularly because the VOCs produced by bacteria and fungi at any given time seems to depend on the VOC signals they are receiving. Except for a single *Trichoderma* sp. isolate from cultivated cranberry bogs, we have not examined the effect of bVOCs on “beneficial” fungi in the soil, such as ericoid mycorrhizal fungi, which are critical for nitrogen and phosphorous nutrition in cranberry (Kosola et al., 2007; Martin and Nehls, 2009). These studies remain for the future.

## Conclusions

We investigated VOCs produced by bacteria isolated from the rough-and-tumble competitive environment of soil as potential biological control agents of fungi. Our finding that *C. vaccinii* produces VOCs that can inhibit fungal growth by more than 80% holds the potential for controlling fungal growth in a wide range of applications. One of the most significant findings from this study is that the co-cultures of bacteria and fungi have emergent volatile metabolome properties. That is, the metabolome of the co-culture cannot be predicted by the sum of its constituents in monoculture. Undoubtedly the metabolomes we detected in co-culture will not fully extrapolate to more complex systems involving VOCs from plants and other plant and soil-associated microbes. Hence, a systems biology approach is required to characterize and ultimately predict the emergent properties of microbiomes in natural and engineered systems, and to discover VOCs for safe and effective control of plant, human, and animal mycoses.

## Data Availability Statement

The datasets generated for this study can be found in the NCBI: MT150599, MT150598, MT227805, MT150597, MT101734, MT101742, MT101743, MT101740, MT101737, MT101748, MT101735, MT101733, MT101747, MT101738, MT101745, MT101739, MT101744, MT101741, MT101736, MT101746, MT158224, MT215537.

## Author Contributions

SS and HB conceived of the project. HB and GE designed the experiments. GE and EH collected and analyzed the data. All authors wrote and edited the manuscript and approved the final version.

## Conflict of Interest

The authors declare that the research was conducted in the absence of any commercial or financial relationships that could be construed as a potential conflict of interest.
