# Supplementary material for: Inhibition of Fungal Growth and Induction of a Novel Volatilome in Response to Chromobacterium vaccinii Volatile Organic Compounds
Source: Front Microbiol. 2020 May 20;11:1035. doi: 10.3389/fmicb.2020.01035 (PMC7251293; doi:10.3389/fmicb.2020.01035)
Supplement: Supplementary file 1 [file Data_Sheet_1.pdf]

Supplementary Information for

Inhibition of Fungal Growth and Induction of a Novel Volatilome in Response to *Chromobacterium vaccinii* Volatile Organic Compounds

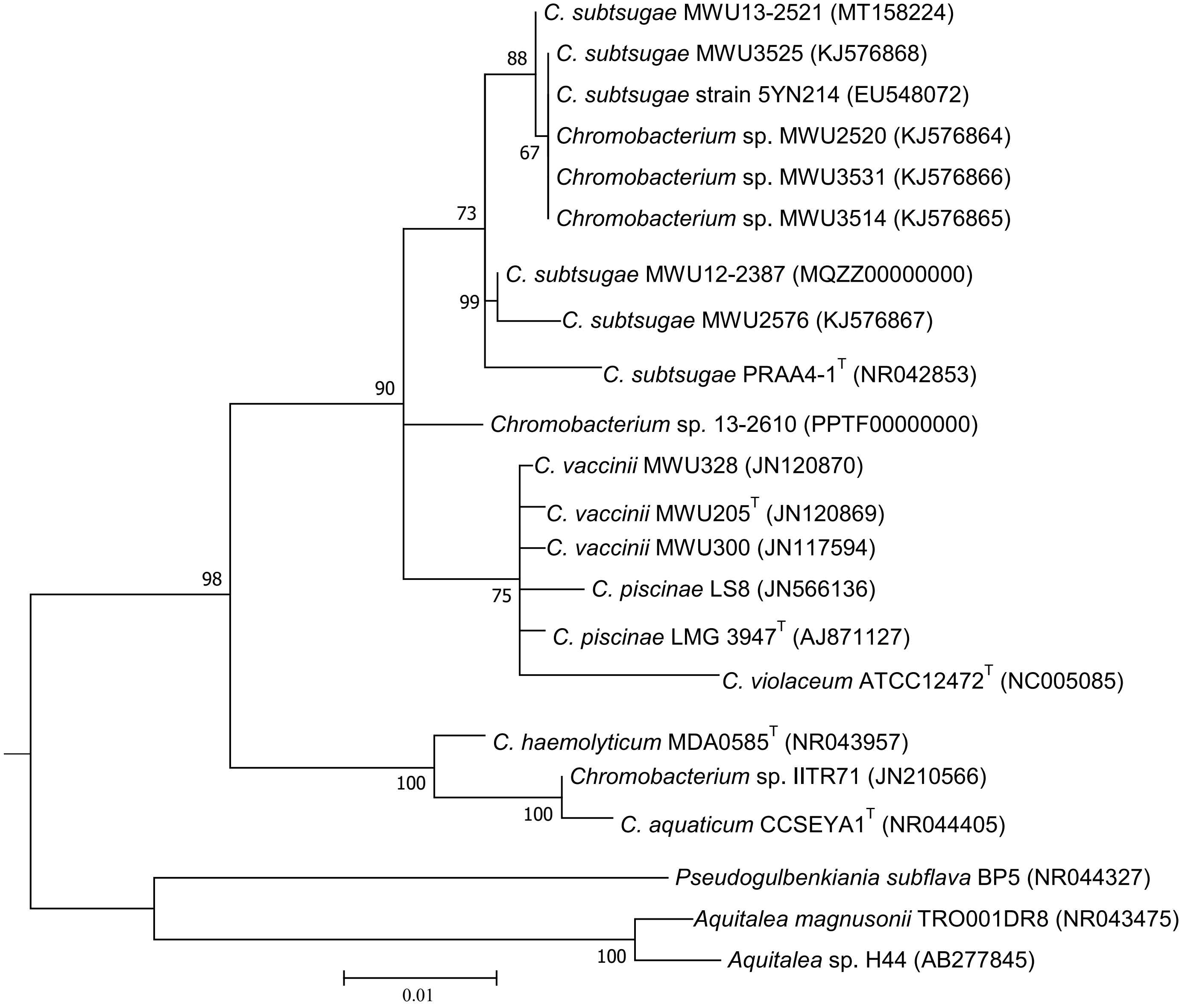

Supplementary Figure 1. Primary *Chromobacterium* clades by 16S rRNA sequence. A Molecular Phylogeny of the genus *Chromobacterium* was inferred using Maximum Likelihood showing *C. subtsugae* MWU12-2387 and *C. subtsugae* MWU13-2521 in two subclades, but both unambiguously in the *Chromobacterium subtsugae* clade. Tree construction is based on the Hasegawa-Kishino-Yano model (Hasegawa et al., 1985) with discrete Gamma distribution (5 categories (+G, parameter = 0.1000)). This model allows for some sites to be evolutionarily invariable ([+I], 78.27% sites). The tree with the highest log likelihood (-3124.18) is shown with bootstrap values given next to the branches based on 500 iterations. Initial tree(s) for the heuristic search were obtained automatically by applying Neighbor-Join and BioNJ algorithms to a matrix of pairwise distances estimated using Maximum Composite Likelihood, and then selecting the topology with highest log likelihood. The tree is drawn to scale, with branch lengths measured in the number of substitutions per site. The analysis involved 24 nucleotide sequences with complete deletion of apparent indels. There are 1280 positions in the final dataset. Evolutionary analyses were conducted in MEGA7 (Kumar et al., 2016). Superscript T (^{T}) indicates the type isolate for the species.

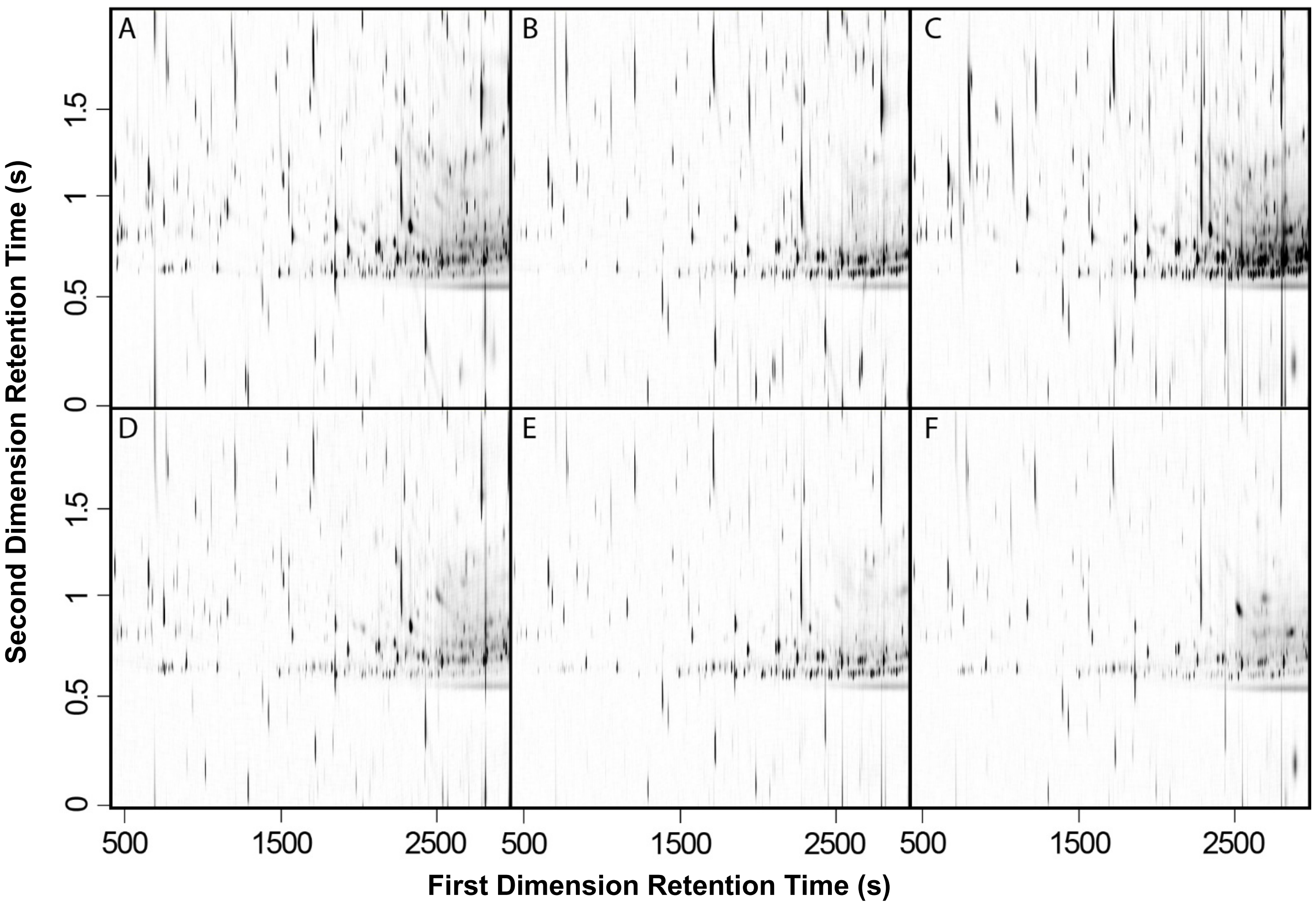

Supplementary Figure 2: Representative GC×GC chromatograms of the volatile metabolomes of *C. vaccinii* MWU328 + *Phoma* sp. (A), *C. vaccinii* MWU328W + *Phoma* sp. (B), *Phoma* sp. (C), *C. vaccinii* MWU328 (D), *C. vaccinii* MWU328W (E), Media control (F). Chromatographic features with first dimension retention times < 358 s were removed from statistical analyses, and the correlated chromatographic areas have been removed for visual clarity.

Supplementary Figure 3: Interactive three dimensional rendering of the principal component analysis score plot (Figure 5) of mono-cultures of *Phoma* sp. (red), *C. vaccinii* MWU328 (yellow), and *C. vaccinii* QS mutant MWU328W (blue), and co-cultures of *Phoma* sp. and MWU328 (orange) and *Phoma* sp. and MWU328W (purple), based upon 53 biogenic volatile metabolites. Six biological replicates were performed for each experiment. (See HTML file provided as a separate file).

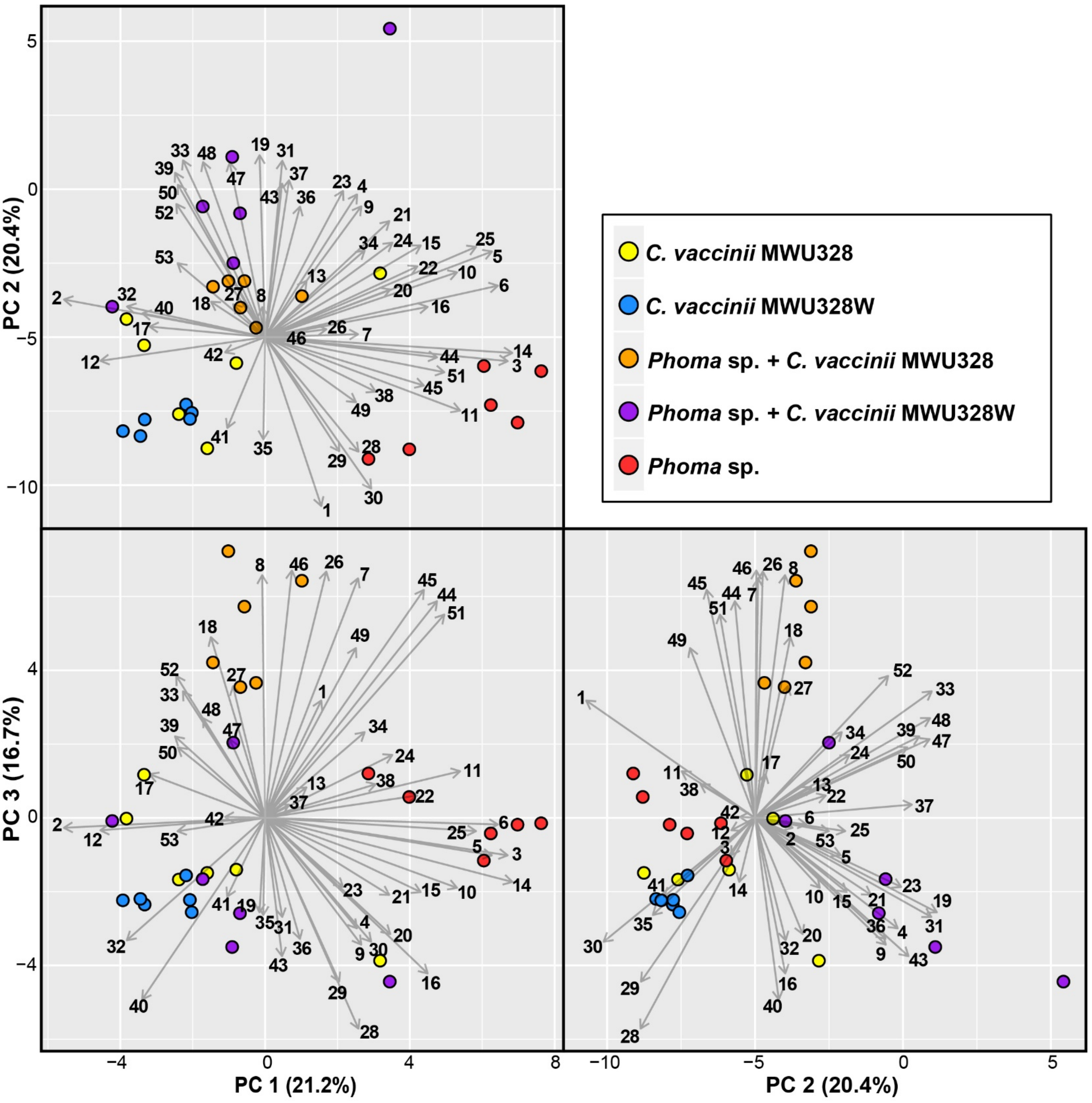

Supplementary Figure 4: Principal component analysis biplots of mono-cultures of *Phoma* (red), MWU328 (yellow), and MWU328W (blue), and co-cultures of *Phoma* and MWU328 (orange) and *Phoma* and MWU328W (purple), based upon 53 biogenic volatile metabolites. Six biological replicates were performed for each experiment.

Supplementary Table 1: Bacterial and fungal strains used in this study. Bacterial strain numbers other than *P. chlororaphis* and *C. vaccinii* are formatted as MWUYY-####, where YY indicates a two-year date for the year of isolation, and the first digit after the hyphen (or the first numeral in the *C. vaccinii* strain names) indicates collection from wild cranberry bogs in the Cape Cod National Seashore (beginning with the digit '2') or a commercial cranberry bog (beginning with the digit '3').

Strain ID	Accession Number	Reference
**Bacteria**		
Pseudomonas chlororaphis 30-84	AHHJ00000000	(Yu et al., 2018)
Bacillus thuringiensis MWU12-2420	MT101734	This work
Bacillus cereus MWU14-2326	MT101742	This work
Aquitalea sp. MWU14-2410	MT101743	This work
Pseudomonas sp. MWU13-2590	MT101740	This work
Pseudomonas sp. MWU12-2517	MT101737	This work
Pseudomonas sp. MWU15-20650	MT101748	This work
Burkholderia cepacia MWU13-2092	MT101735	This work
Burkholderia tropica MWU12-2056	MT101733	This work
Xylophilus ampelinus MWU14-20187	MT101747	This work
Acinetobacter calcoaceticus MWU13-2536	MT101738	This work
Ewingella americana MWU14-20116	MT101745	This work
Serratia marcescens MWU13-2543	MT101739	This work
Lysinibacillus sp. MWU14-2414	MT101744	This work
Paenibacillus sp. MWU13-2602	MT101741	This work
Enterobacter sp. MWU13-2507	MT101736	This work
Delftia sp. MWU13-3324	MT101746	This work
Chromobacterium subtsugae MWU13-2521	MT158224	This work
Chromobacterium subtsugae MWU12-2387	LCWR00000000	(Voing et al., 2017)
Chromobacterium vaccinii MWU205	JN120869	(Soby et al., 2013)
Chromobacterium vaccinii MWU300	JN117594	(Soby et al., 2013)
Chromobacterium vaccinii MWU328	JN120870	(Soby et al., 2013)
Chromobacterium vaccinii MWU328W (*cviR*$^{-}$)	MT215537	This work
**Fungi**		
Trichoderma sp. MWU14-9201	MT150599	This work
Phoma sp. MWU-UMCS9302	MT150598	This work
Colletotrichum sp. MWU-UMCS9301	MT227805	This work
Coleophoma sp. MWU-UMCS9305	MT150597	This work
**Oomycete**		
Phytophthora cinnamomi R001	MG560190	(Weiland et al., 2018)

Supplementary Table 2: Compound identification, chromatographic characteristics, and mean peak intensities for the 53 VOCs that differ significantly in abundance between microbial cultures and media controls. (See Excel spreadsheet provided as a separate file)

Supplementary Table 3: Volatile metabolites that are produced by MWU328 and/or MWU328W. Metabolites in blue or yellow are more abundant in the mutant or wild-type, respectively ($p < 0.1$).

**Compound ID**	**Fold Change**	***p*- value**
Thiazole	6.28×10^{-1}	0.23
Dimethyl disulfide	4.38×10^{-1}	0.04
UNK-3	7.33×10^{1}	0.17
CA-4	5.41×10^{0}	0.21
CA-5	1.41×10^{3}	0.18
UNK-6	1.59×10^{3}	0.18
HC-8	7.43×10^{0}	0.18
O-9	4.48×10^{2}	0.18
1,2-Ethanediol, diacetate	2.46×10^{2}	0.10
S,N-11	6.59×10^{-1}	0.36
Dimethyl trisulfide	1.09×10^{0}	0.45
N-13	1.76×10^{-3}	0.09
CA-14	4.14×10^{2}	0.18
N-15	3.98×10^{2}	0.18
EST-16	7.90×10^{-1}	0.43
1-Octanol	6.47×10^{0}	0.02
CA-18	8.62×10^{3}	0.01
UNK-20	5.98×10^{-1}	0.30
UNK-21	3.36×10^{0}	0.18
UNK-22	7.28×10^{2}	0.11
ETH-23	1.83×10^{1}	0.16
Octanoic acid	5.70×10^{0}	0.04
CA-25	1.43×10^{1}	0.18
UNK-27	1.17×10^{4}	0.08
UNK-28	2.20×10^{0}	0.15
ETH-29	3.66×10^{0}	0.16
ETH-30	2.29×10^{-1}	0.04
Indole	9.46×10^{-5}	0.04
EST-33	2.22×10^{1}	0.11
EST-34	2.59×10^{0}	0.01
UNK-35	9.08×10^{-1}	0.47
UNK-36	1.48×10^{3}	0.18
EST-38	1.18×10^{2}	0.14
ETH-39	4.67×10^{1}	0.17
T-40	1.05×10^{0}	0.48
UNK-41	1.74×10^{0}	0.24
N-42	1.97×10^{3}	0.11
UNK-43	2.99×10^{2}	0.18
ARO-45	8.84×10^{0}	0.18
UNK-47	9.80×10^{2}	0.18
EST-48	5.26×10^{-1}	0.26
UNK-49	7.97×10^{-1}	0.37
ETH-50	2.42×10^{1}	0.13
UNK-52	7.18×10^{0}	0.20
N-53	6.00×10^{-1}	0.36

References

Hasegawa, M., Kishino, H., and Yano, T.A. (1985). Dating of the human ape splitting by a molecular clock of mitochondrial-DNA. J. Mol. Evol. 22**,** 160-174.

Kumar, S., Stecher, G., and Tamura, K. (2016). MEGA7: Molecular evolutionary genetics analysis version 7.0 for bigger datasets. Mol. Biol. Evol. 33**,** 1870-1874.

Soby, S.D., Gadagkar, S.R., Contreras, C., and Caruso, F.L. (2013). *Chromobacterium vaccinii* sp. nov., isolated from native and cultivated cranberry (*Vaccinium macrocarpon* Ait.) bogs and irrigation ponds. Int. J. Syst. Evol. Microbiol. 63**,** 1840-1846.

Voing, K., Harrison, A., and Soby, S.D. (2017). Draft genome sequence of *Chromobacterium subtsugae* MWU12-2387 isolated from a wild cranberry bog in Truro, Massachusetts. Microbiol. Resour. Announc. 5.

Weiland, J.E., Scagel, C.F., Grunwald, N.J., Davis, E.A., Beck, B.R., and Fieland, V.J. (2018). Variation in disease severity caused by *Phytophthora cinnamomi*, *P. plurivora*, and *Pythium cryptoirregulare* on two rhododendron cultivars. Plant Dis. 102**,** 2560-2570.

Yu, J.M., Wang, D., Pierson, L.S., 3rd, and Pierson, E.A. (2018). Effect of producing different phenazines on bacterial fitness and biological control in *Pseudomonas chlororaphis* 30-84. Plant Pathol. J. 34**,** 44-58.
